# Ameliorative Effect of Saffron Aqueous Extract on Hyperglycemia, Hyperlipidemia, and Oxidative Stress on Diabetic Encephalopathy in Streptozotocin Induced Experimental Diabetes Mellitus

**DOI:** 10.1155/2014/920857

**Published:** 2014-07-09

**Authors:** Saeed Samarghandian, Mohsen Azimi-Nezhad, Fariborz Samini

**Affiliations:** ^1^Department of Basic Medical Sciences, Neyshabur University of Medical Sciences, Neyshabur 9314634814, Iran; ^2^Health Strategic Research Center, Neyshabur University of Medical Sciences, Neyshabur, Iran; ^3^Department of Genetics, Faculty of Medicine, Mashhad University of Medical Sciences, Mashhad, Iran; ^4^Department of Neurosurgery, Faculty of Medicine, Mashhad University of Medical Sciences, Mashhad, Iran

## Abstract

Diabetic encephalopathy is one of the severe complications in patients with diabetes mellitus. Findings indicate that saffron extract has antioxidant properties but its underlying beneficial effects on diabetic encephalopathy were unclear. In the present study, the protective activities of saffron were evaluated in diabetic encephalopathy. Saffron at 40 and 80 mg/kg significantly increased body weight and serum TNF-*α* and decreased blood glucose levels, glycosylated serum proteins, and serum advanced glycation endproducts (AGEs) levels. Furthermore, significant increase in HDL and decrease (*P* < 0.05) in cholesterol, triglyceride, and LDL were observed after 28 days of treatment. At the end of experiments, the hippocampus tissue was used for determination of glutathione content (GSH), superoxide dismutase (SOD), and catalase (CAT) activities. Furthermore, saffron significantly increased GSH, SOD, and CAT but remarkably decreased cognitive deficit, serum TNF-*α*, and induced nitric oxide synthase (iNOS) activity in hippocampus tissue. Our findings indicated that saffron extract may reduce hyperglycemia and hyperlipidemia risk and also reduce the oxidative stress in diabetic encephalopathy rats. This study suggested that saffron extract might be a promising candidate for the improvement of chemically induced diabetes and its complications.

## 1. Introduction

Diabetes mellitus is a serious metabolic disorder which is a major source of ill health all over the world and its incidence is expected to increase by 5.4% in 2025 [1]. It has been shown that patients with diabetes mellitus have increased oxidative stress and impaired antioxidant defense systems, which appear to contribute to the initiation and progression of diabetes-associated complications [2]. Diabetic encephalopathy has been recognized as a severe diabetic complication, with adverse functional and cognitive effects. There is a growing body of evidence to support the assertion that the deficits are associated with the damage to the hippocampal neurons [3]. The increased mortality and morbility of encephalopathy in patients with type 2 diabetes is closely associated with hyperglycemia, hyperinsulinemia, hypercholesterolemia, and hypertension [4]. Advanced glycation endproducts (AGEs), generated by nonenzymatic glycation and oxidation of protein and reducing sugars, are known to initiate and aggravate the pathological damage in diabetic encephalopathy [5]. The accumulation of AGEs can damage brain by eliciting oxidative stress which have been implicated in the damage of the structure and function of the brain's neurons and hippocampus [6]. Therefore, interfering with hyperglycemia, hyperlipidemia, oxidative stress, and the AGEs signaling pathway might have the potential to attenuate hyperglycemia-induced brain damage.

Ethnobotanical field studies revealed that a number of plant remedies were used to alleviate the symptoms of diabetes. Saffron obtained from the pistils of* Crocus sativus* L. flowers and represents the red dry stigmas of the flower. Saffron is used in the traditional medicine for the treatment of numerous diseases including depression, inflammation, cognitive disorders, seizures, and cancer [7, 8]. Further, saffron by its compounds crocin, crocetin, and safranal has high antioxidant activities [9, 10]. The accumulating evidence in vitro and in vivo showed that saffron extract has a beneficial neuroprotective activity on brain ischemia in the rats. Saffron can attenuate the neurotoxicity for the brain's neurons in cerebral infarct and neurodeficit in rat [11].

Diabetes-linked alterations in antioxidant defense system enzymes, such as catalase, glutathione peroxidase, and superoxide dismutase, have been demonstrated [2]. The negative impact of diabetes on the retinal, renal, nervous, and cardiovascular systems is well recognized, yet little is known about its effect on the brain. On the other hand, whether saffron extract has hypoglycemic, hyperlipidemia, and antioxidant activities that can prevent the pathological damage of diabetes in STZ-induced diabetic rats is unknown. Thus, the present study was designed to evaluate the effect of saffron extract on a model of diabetes mellitus and its effects on serum lipid profiles, tumor necrosis factor-alpha (TNF-*α*), a marker for inflammation, and oxidative stress parameters in this process and the other aim of the present investigation was to evaluate the cognitive function and hypoglycemic and antioxidant activities of saffron extract on diabetic encephalopathy in STZ-induced rats.

## 2. Materials and Methods

### 2.1. Reagents

All purified enzymes, coenzymes, substrates, standards, buffers, kits, and the other chemicals were purchased from Sigma-Aldrich Chemical (St. Louis, USA).

### 2.2. Preparation of Aqueous Extract of Saffron

The saffron used in this study was collected from a private garden and identified by botanists in the herbarium of Ferdowsi University of Mashhad (specimen number 293-0303-1). The part of* C. sativus* that is being used as additive and also as herbal medicine is the stigma. The pistils of saffron flowers were air-dried in the shade before extraction. The stigmas were being powdered for 10 min with the use of mortar and pistil. After grinding, aqueous extract was prepared with 15 g of its ground stigma and 400 mL of distilled water in a Soxhlet extractor for 18 hours. The prepared extract was concentrated to 100 mL with a rotatory evaporator in low pressure, then the extract was dried at 35°C–40°C, and the yield of extraction was 25 mg of freeze-dried powder per 100 mg dry stigma and the extraction was repeated five times. Finally, the extract was filtered through a 0.2 mm filter to be sterilized. The resultant solution was stored at 4°C to 8°C. To prepare the solutions for injection, after weighing, powder was dissolved in distilled water and immediately administered to the animals. The high-performance liquid chromatography (HPLC) analysis of saffron extract using UV detector with wavelength of 308 nm showed the presence of safranal with concentration of 0.514% and also HPLC analysis of saffron extract using UV detector with wavelength of 443 and 250 nm showed the presence of all crocins and picrocrocin with concentration of 95.83% and 3.02%, respectively.

### 2.3. Study Design

50 male Wistar albino rats weighing 180–220 g were randomly allotted to five experimental groups (*n* = 10 per group) as follows. The animals were kept in their own cages in a controlled environment, with constant room temperature (21 ± 2°C) under a normal 12 h light/dark cycle. Standard food and water were given ad libitum. Rats in Group A were fed with standard feed and received intraperitoneal (i.p.) injection of 0.9% saline for blank control (C). The animals were housed according to regulations for the Welfare of Experimented Animals. The study was conducted in Mashhad Medical University Experimental Animal Research Laboratory. Protocols were approved by the Ethical Committee (The Ethical Research Committee of Mashhad University of Medical Sciences). On the first day of the study, the diabetic groups were given streptozocin in a single intraperitoneal (i.p.) injection at a dose of 60 mg/kg for induction of diabetes. Blood was extracted from the tail vein for glucose analysis 72 hours after streptozocin injection. Rats with blood glucose levels greater than 250 mg/dL were considered as diabetic and used for the study. Three days after the induction of diabetes, STZ-treated rats were divided into four groups of ten animals. Group B was left without treatment and served as the untreated diabetic group (D). Groups C, D, and E were treated with saffron extract (i.p. injection) at a daily dose of 20, 40, and 80 mg/kg, respectively. These injections were continued to the end of the study (4 weeks). The daily dosages of saffron extract were used in this experiment and were estimated from the published literature [12]. Groups included low-saffron group (L-saf), moderate-saffron group (M-saf), and high-saffron group (H-saf).

Blood glucose level and body weights were recorded at weekly intervals. At the end of the 4-week period, animals were killed by pentobarbital overdose (150 mg/kg, i.p.), and blood was subsequently collected from the retroorbital sinus. Blood and sera were separated by centrifugation at 3000 rpm for 10 min and stored at −80°C until determination within 12 h for glucose and lipid profile. After the rats were sacrificed, brains were removed carefully and then the hippocampi were immediately dissected on a cold plate, weighed, and homogenized with ice cold saline. The homogenate was centrifuged at 3000 g for 10 min at 4°C. Finally, the supernatant was taken for use.

### 2.4. Morris Water Maze Test

Animals were tested in the Morris water maze test [13]. The apparatus consisted of a circular water tank. A platform invisible to the rats was set inside the tank and filled with water maintained at approximately 28 ± 2°C. The tank was located in a large room where there were several brightly colored cues external to the maze; these were visible from the pool and could be used by the rats for spatial orientation. The position of the cues remained unchanged throughout the study. The water maze task was carried out for 5 consecutive days. The rats received four consecutive daily training trials in the following 5 days, with each trial having a ceiling time of 90 s and a trial interval of approximately 30 s. For each trail, each rat was put into the water at one of four starting positions, the sequence of which being selected randomly. During test trials, rats were placed into the tank at the same starting point, with their heads facing the wall. The rat had to swim until it climbed onto the platform submerged underneath the water. After climbing onto the platform, the animal remained there for 20 s before the commencement of the next trial. The escape platform was kept in the same position relative to the distal cues. If the rat failed to reach the escape platform within the maximally allowed time of 90 s, it was gently placed on the platform and allowed to remain there for the same amount of time. The time to reach the platform (latency in seconds) was measured.

### 2.5. Measurement of Blood Glucose

Glucose concentrations were measured with the Ames One Touch glucometer (One-Touch Basic; Lifescan, Johnson and Johnson, New Brunswick, NJ) in rat tail vein blood. Blood glucose was estimated using the diagnostic kits (Pars Azmoon kit, IRI) on an automatic analyzer (Abbott, model Alcyon 300, USA).

### 2.6. Estimation of Tumor Necrosis Factor-Alpha (TNF-*α*)

Tumor necrosis factor-alpha (TNF-*α*) was estimated using rat TNF-*α* kit (R&D Systems). It is a solid phase sandwich enzyme linked immunosorbent assay (ELISA) using a microtitre plate reader at 450 nm. Concentrations of TNF-*α* were calculated from plotted standard curve. TNF-*α* levels were expressed as mean ± SEM.

### 2.7. Measurement of Serum Lipid Profile

The concentrations of total lipids, triglycerides, total cholesterol, low-density lipoprotein (LDL) cholesterol, and high-density lipoprotein (HDL) cholesterol in serum were estimated by using diagnostic kits (Pars Azmoon kit, IRI) on an automatic analyzer (Abbott, model Alcyon 300, USA).

### 2.8. Measurement for Glycosylated Serum Proteins in Serum

Serum concentrations of glycosylated serum proteins (GSP) were detected using a commercial kit. In brief, an aliquot of serum (100 *μ*L) was mixed with 2 mL of nitroblue tetrazolium maintained at 37°C for 15 min. At the end of the reaction, a stabilizer was added to the mixture. Finally, the absorbance of the mixture was read at 530 nm with a spectrophotometer.

### 2.9. Enzyme-Linked Immunosorbent Assay for AGEs in Serum

Enzyme-linked immunosorbent assay was performed to quantity AGEs concentration in serum according to the reported method [14]. Briefly, 96-well plate was incubated with diluted serum sample (1 : 5) and standard AGE-bovine serum albumin (40, 80, 160, 320, and 480 ng/L) for 30 min at 37°C. An aliquot of phosphate buffered saline (PBS) was used for the blank control and then the wells were washed with a wash buffer for five times. Horseradish peroxidase- (HRP-) labeled AGEs antibody (50 *μ*L) was added to maintain for another 30 min at 37°C. After being washed five more times, the plate was incubated with 100 *μ*L of chromogenic agent tetramethyl benzidine with a dark environment for 30 min at 37°C. The blue reaction was changed to yellow by an addition of 50 *μ*L acidic stopping solution. Finally, the absorbance of the samples was measured with a spectrophotometer at 450 nm.

### 2.10. Preparation of Test Samples of Hippocampus

The hippocampus tissue of rats was taken quickly after being sacrificed and was excised quickly under sterile conditions. The tissue was cut into pieces and then added with cold saline containing 1% Triton X-100 to prepare the homogenate of hippocampus tissue. The samples were then collected and centrifuged at 14,000 g at 4°C. The supernatant fraction was used for estimation of the glutathione (GSH) content, SOD, CAT activities, and induced nitric oxide synthase (iNOS) activity assays within 6 h. Whole homogenates were used for measurement of lipid peroxidation.

### 2.11. Assay of GSH Content in Hippocampus Tissue

The GSH content in hippocampus homogenate was determined as described previously [15] and expressed as *μ*mol/L.

### 2.12. Assay of SOD

The activity of SOD was determined by the method of S. Marklund and G. Marklund (1974), using inhibition of pyrogallol autooxidation at Ph 8.0 [16]. The specific activity of SOD is expressed as units per mg protein.

### 2.13. Catalase Activity Assay

Catalase activity was assayed by H_2_O_2_ consumption, following Aebi's (1984) [17]. Briefly, The decomposition of H_2_O_2_ can be followed directly by measuring the decrease in absorbance at 240 nm. The difference in absorbance per unit time is a measure of CAT activity. Previously, hippocampus homogenate aliquots were centrifuged at 1000 g and 4°C for 10 min. The adequate amount of supernatants (60 *μ*L equivalent to 1.5 mg tissue wet weight) was added to a reaction mixture that contained 0.002% Triton X-100, 0.1 mm EDTA, 0.5 m phosphate buffer, pH = 7.0, and 15 mm H_2_O_2_ in 1 mL final volume. Activity was calculated with the initial 30 s decomposition rate and the results are expressed in mmol/mg protein.

### 2.14. Assay for iNOS Activity in Hippocampus Tissue

The pathogenesis of diabetic encephalopathy is associated with oxidative stress in the body; therefore, the activity iNOS in the serum was measured. The activity of iNOS in the serum was measured by an iNOS activity assay kit according to the manufacturer's instructions (Beyotime Institute of Biotechnology) using a fluorescence microplate reader with excitation/emission wavelength of 495/515 nm.

### 2.15. Analysis of Body Weight during Treatment Period

Body weight was recorded at weekly intervals in all the experimental groups.

### 2.16. Statistical Analysis

Data from the three groups were expressed as means ± standard deviation. The statistical analysis was performed by one-way analysis of variance using GraphPad Prism 5.0 statistical software. Differences between treatment groups were analyzed by Tukey's test. *P* < 0.05 was considered statistically significant.

## 3. Results

### 3.1. Saffron Extract Significantly Increased Body Weight in STZ-Induced Diabetic Rats

As shown in Table 1, during the experimental period (4 weeks), STZ induced diabetic rats had significantly lower body weights (183 ± 11 g) compared with the blank control (301 ± 18 g). However, the treatment with 20 mg/kg saffron extract prevented STZ-decreased body weight in diabetic rats (194 ± 15 g). Interestingly, the treatment with M-saf (40 mg/kg) and H-saf (80 mg/kg) also significantly increased body weight in diabetic rats (231 ± 12 g and 239 ± 14 g, resp.). Furthermore, saffron extract increased the body weight in a dose-dependent manner (Table 1). The results indicate that saffron could improve the body weight in diabetic rats.

### 3.2. Saffron Extract Significantly Decreased Blood Glucose Levels

**Figure 1:**
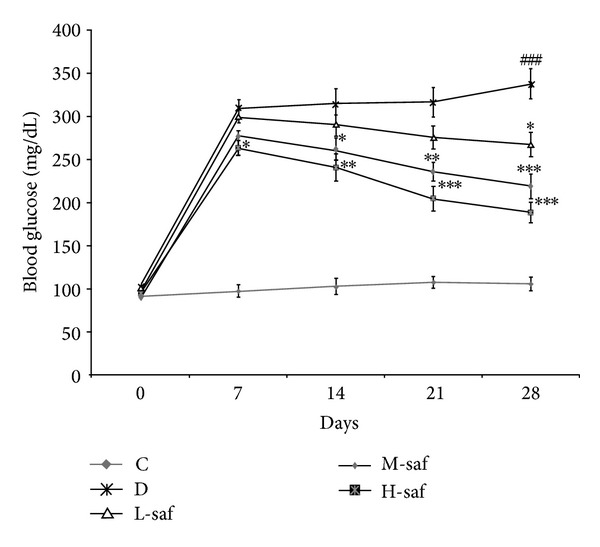
Effect of saffron extract on blood glucose level (mg/dL). Control (C), untreated diabetic rats (D), saffron extract (20 mg/kg/day)—treated diabetic (L-saf), saffron extract (40 mg/kg/day)—treated diabetic (M-saf) and saffron extract (80 mg/kg/day)—treated diabetic (H-saf) rats during 4 weeks of study (*n* = 10, for each group). Values are the mean ± SEM. Statistical significance for the difference between the data of the control group versus other groups: ^###^ *P* < 0.001. Statistical significance for the difference between the data of untreated diabetic group versus treated groups: *P < 0.05, **P < 0.01, ***P < 0.001.

The diabetic rats exhibited significant (*P* < 0.001) hyperglycemia compared with the control rats (Figure 1). After 4 weeks the saffron extract dose dependently decreased blood glucose levels in the diabetic rats compared with the untreated diabetic rats (Figure 1). Saffron extract (20 mg/kg/day) significantly decreased glucose in STZ diabetic rats only at the 4th week of the study (*P* < 0.05), while at 40 mg/kg/day saffron reduced significantly blood glucose significantly at the 2nd, 3rd, and 4th week from induction of diabetes compared with untreated diabetic rats (*P* < 0.05, *P* < 0.01, and *P* < 0.001, resp.). At the highest dose of saffron extract (80 mg/kg/day), serum blood glucose was significantly reduced beginning from the first week of the treatment (*P* < 0.05) (Figure 1).

### 3.3. Effect of Saffron Extract on Diabetes-Induced Cognitive Deficit

**Figure 2:**
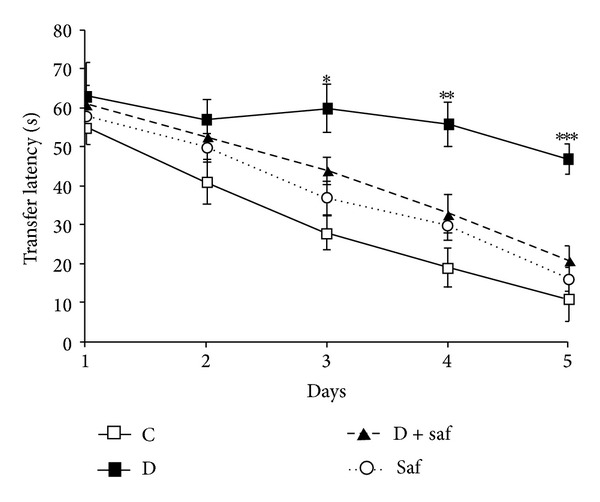
Effect of saffron treatment on the performance of spatial memory acquisition phase in diabetic rats. Data are expressed as mean ± S.E.M. *P < 0.05, **P < 0.01, ***P < 0.001 and different from control and saffron groups from the 3rd day of the training sessions.

The cognitive function was assessed in the Morris water maze test. The mean escape latency for the trained rats decreased from 55 to 11 s over the course of the 20 learning trials. The mean escape latency did not differ between any of the groups on the first and second days of testing in Morris water maze but from the third day onwards there was significant difference in transfer latency between diabetic (60 ± 6.1) and control (28 ± 4.3) animals. Treatment of saffron significantly decreased mean transfer latency in diabetic animals (Figure 2). Diabetic animals showed a lower ability to find the platform and learn its location in the 5th day of training. This poorer performance was prevented by the treatment with saffron and decreased latency to find the platform from the 3rd day of training (*P* < 0.05).

### 3.4. Effect of Saffron on Tumor Necrosis Factor-Alpha (TNF-*α*)

**Figure 3:**
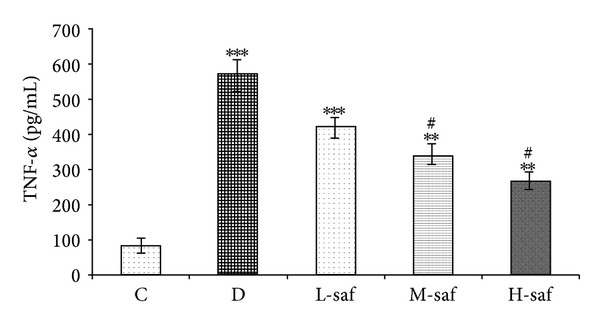
Effect of saffron extract on tumor necrosis factor-alpha (TNF-*α*). Serum TNF-*α* level was markedly increased in diabetic rats as compared to control. Saffron resulted in significant decreased serum TNF-*α* level.

Serum TNF-*α* level was markedly (*P* < 0.001) increased (571.91 ± 51.28 pg/mL) in diabetic rats as compared to control (83.5 ± 14.7 pg/mL). Interestingly, the serum TNF-*α* level decreased significantly (*P* < 0.05) to 340.4 ± 27.9 and 265.8 ± 21.4 pg/mL after the administration of 40 and 80 mg/kg saffron extract, respectively (M & H-saf versus untreated diabetic group) (Figure 3).

### 3.5. Saffron Extract Significantly Ameliorate Serum Lipid Profile Levels

**Figure 4:**
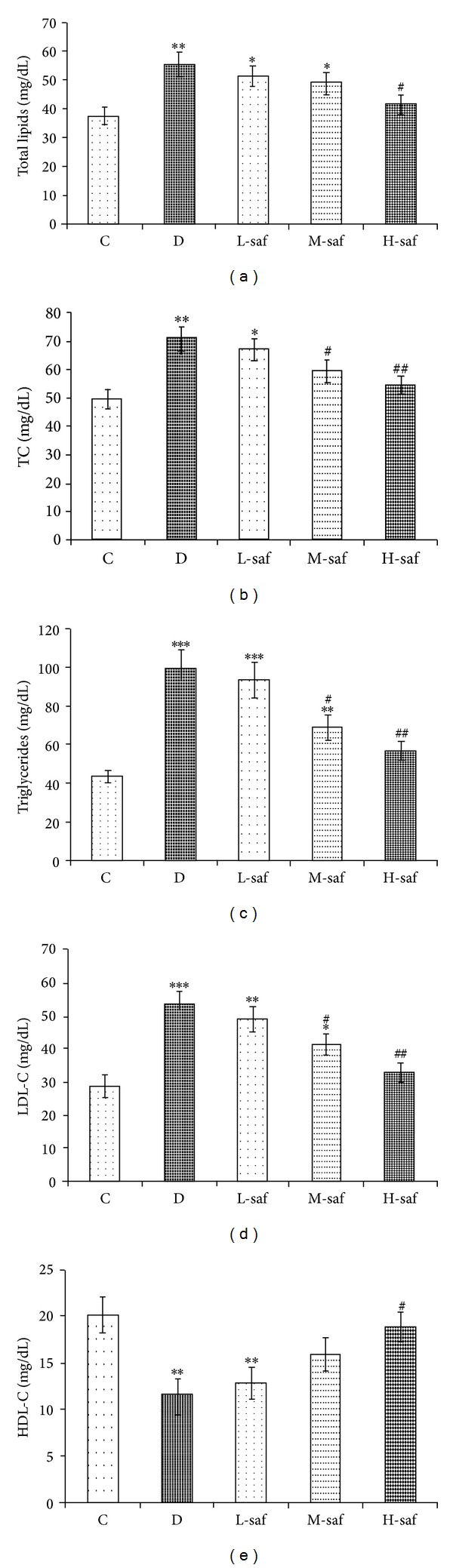
Effect of saffron extract on plasma lipid profiles (mg/dL) (a) total lipid, (b) total cholesterol (TC), (c) triglycerides, (d) LDL-C, (e) HDL-C, in control (C), untreated diabetic rats (D), saffron extract (20 mg/kg/day)—treated diabetic (L-saf), saffron extract (40 mg/kg/day)—treated diabetic (M-saf) and saffron extract (80 mg/kg/day)—treated diabetic (H-saf) rats during 4 weeks of study (*n* = 10, for each group). Values are the mean ± SEM. Statistical significance for the difference between the data of the control group versus other groups: *P < 0.05, **P < 0.01, ***P < 0.001. Statistical significance for the difference between the data of untreated diabetic group versus treated groups: ^#^ *P* < 0.05, ^##^ *P* < 0.01.

STZ-injected rats showed significant increases in the serum levels of total lipids, triglycerides, total cholesterol, and LDL but significantly decreased serum HDL level compared to the control group (Figure 2). Saffron extract dose dependently reduced the serum levels of total lipids, triglycerides, total cholesterol, and LDL but, however, increased serum HDL level during the experimental period. At the highest saffron extract dose (80 mg/kg/day) there was no significant difference in total lipid, cholesterol, LDL, and HDL levels between the STZ-treated rats and the control rats (Figure 4).

### 3.6. Effects of Saffron Extract on GSP

**Figure 5:**
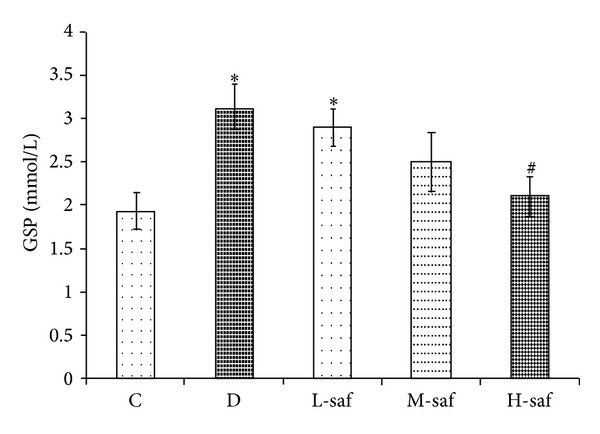
Effect of saffron extract on glycosylated serum proteins (GSP) (mmol/L) in control (C), untreated diabetic rats (D), saffron extract (20 mg/kg/day)—treated diabetic (L-saf), saffron extract (40 mg/kg/day)—treated diabetic (M-saf) and saffron extract (80 mg/kg/day)—treated diabetic (H-saf) rats during 4 weeks of study (*n* = 10, for each group). Values are the mean ± SEM. Statistical significance for the difference between the data of the control group versus other groups: *P < 0.05. Statistical significance for the difference between the data of untreated diabetic group versus treated groups: ^#^ *P* < 0.05.

As shown in Figure 3, there was a significant increase in GSP level between the untreated diabetic group (3.10 ± 0.35 mmol/L) and control (1.93 ± 0.21 mmol/L) (*P* < 0.05). The 20, 40, and 80 mg/mL doses of saffron extract decreased GSP levels up to 6.45% (2.90 ± 0.21 mmol/L), 19.35% (2.50 ± 0.34 mmol/L), and 32.25% (2.10 ± 0.39 mmol/L), respectively (Figure 5); however, only at the highest dose (80 mg/mL) the treatment significantly decreased GSP levels compared with untreated diabetic group (*P* < 0.05).

### 3.7. Saffron Extract Significantly Decreased Serum AGEs

**Figure 6:**
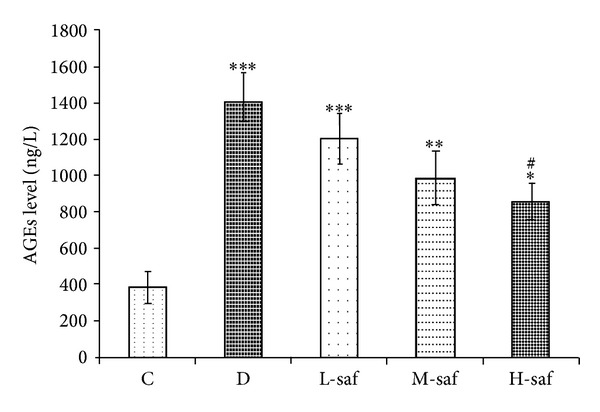
Effect of saffron extract on advanced glycation endproducts (AGEs) (ng/L) in control (C), untreated diabetic rats (D), saffron extract (20 mg/kg/day)—treated diabetic (L-saf), saffron extract (40 mg/kg/day)—treated diabetic (M-saf) and saffron extract (80 mg/kg/day)—treated diabetic (H-saf) rats during 4 weeks of study (*n* = 10, for each group). Values are the mean ± SEM. Statistical significance for the difference between the data of the control group versus other groups: *P < 0.05, **P < 0.01, ***P < 0.001. Statistical significance for the difference between the data of untreated diabetic group versus treated groups: ^#^ *P* < 0.05.

The accumulation of AGEs has been implicated in the pathogenesis of diabetic encephalopathy [18]. In this study, the AGEs level in serum in the untreated diabetic group (1401.7 ± 168.5 ng/L) was elevated significantly as compared with the control (385.3 ± 63.3 ng/L). As shown in Figure 6, L-saf, M-saf, and H-sae significantly inhibited AGE formation up to 14.00%, 29.47%, and 39.30% at doses of 20, 40, and 80 mg/kg, respectively. The AGEs level in serum in the H-saf group was decreased significantly as compared with the untreated diabetic group (*P* < 0.05; Figure 6). The findings demonstrate that saffron extract is able to inhibit AGE formation in vivo.

### 3.8. GSH Content in Hippocampus Tissue

**Figure 7:**
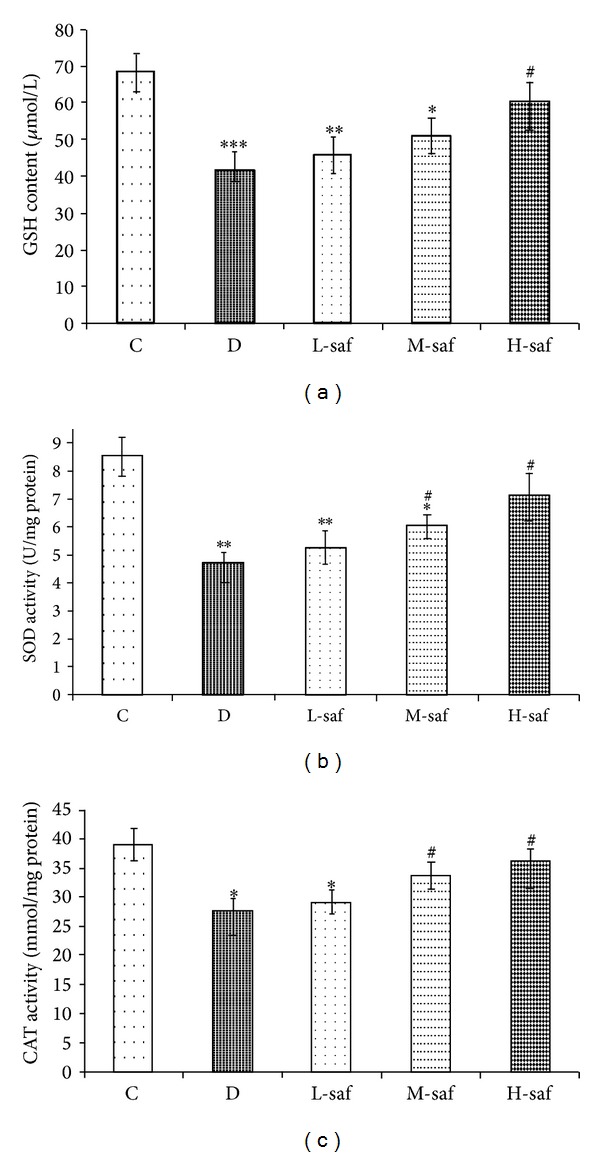
Effect of saffron extract treatment on GSH (a), SOD (b), and CAT levels (c) in hippocampus of diabetic rats. Control (C), untreated diabetic rats (D), saffron extract (20 mg/kg/day)—treated diabetic (L-saf), saffron extract (40 mg/kg/day)—treated diabetic (M-saf) and saffron extract (80 mg/kg/day)—treated diabetic (H-saf) rats during 4 weeks of study (*n* = 10, for each group). Values are the mean ± SEM. Statistical significance for the difference between the data of the control group versus other groups: *P < 0.05, **P < 0.01, ***P < 0.001. Statistical significance for the difference between the data of untreated diabetic group versus treated groups: ^#^ *P* < 0.05.

Oxidative stress plays a crucial role in the development and progression of diabetic encephalopathy. GSH, a small molecule peptide consisting of three amino acids, has been regarded as an important factor in the antioxidant and scavenging of free radicals in vivo. After being induced by 60 mg/kg STZ, the hippocampal GSH content was lower compared with control (41.73 ±4.12 *μ*mol/L versus 68.17 ± 5.21 *μ*mol/L). More importantly, the GSH content in the hippocampus increased significantly to 60.31 ± 5.22 *μ*mol/L after the administration of 80 mg/kg saffron extract (H-saf versus untreated diabetic group) (Figure 7(a)). Our data support the premise that saffron extract has antioxidant activity on the attenuation of memory deficits in diabetic encephalopathy rats.

### 3.9. SOD Content in Hippocampus Tissue

Changes in the activities of SOD in hippocampus of control, untreated diabetic, and saffron extract-treated rats are summarized in Figure 7(b). There was a decrease in SOD in the STZ-diabetic group compared with respective control group (*P* < 0.01). Saffron extract (40 and 80 mg/kg/day) treated diabetic rats significantly increased the SOD content in hippocampus tissue compared with the untreated diabetic rats (*P* < 0.05). In addition, there was not a significant difference between diabetic rats treated with high saffron concentration (80 mg/kg/day) and the control group (Figure 7(b)).

### 3.10. CAT Content in Hippocampus Tissue

There was a decrease in CAT level in the untreated diabetic group compared with respective control group (*P* < 0.05), but CAT activity showed a nonsignificant increase in the rats receiving low concentration of saffron compared with the untreated diabetic group. Supplementation with the moderate and high concentration of saffron for a month (40 and 80 mg/kg/day) significantly increased CAT activities in the hippocampus of diabetic rats versus the untreated diabetic group (*P* < 0.05). In addition, there was not a significant difference between diabetic rats treated with the moderate and high saffron concentrations (40 and 80 mg/kg/day) and the control group (Figure 7(c)). Therefore, treatment with diabetic rats with saffron extract restored the antioxidant enzyme activity nearly to that of control rats.

### 3.11. iNOS Enzyme Activity in Hippocampus Tissue

**Figure 8:**
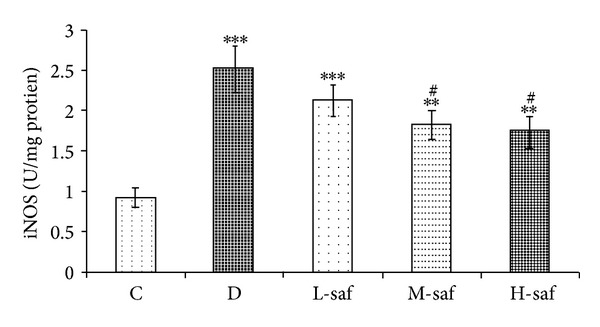
Effect of saffron extract on TNF-alpha release in diabetic rats. Control (C), untreated diabetic rats (D), saffron extract (20 mg/kg/day)—treated diabetic (L-saf), saffron extract (40 mg/kg/day)—treated diabetic (M-saf) and saffron extract (80 mg/kg/day)—treated diabetic (H-saf) rats during 4 weeks of study (*n* = 10, for each group). Values are the mean ± SEM. Statistical significance for the difference between the data of the control group versus other groups: **P < 0.01, ***P < 0.001. Statistical significance for the difference between the data of untreated diabetic group versus treated groups: ^#^ *P* < 0.05.

The NOS isoforms play an important role in hippocampus dependent forms of learning in rats [19]. As shown in Figure 8, STZ markedly enhanced the iNOS level in hippocampus of diabetic rats as compared with the control (2.53 ± 0.28 U/mg protein for untreated diabetes group and 0.92 ± 0.12 U/mg protein for control). Interestingly, the STZ-increased iNOS level in the hippocampus was decreased significantly by the treatment with saffron extract of 40 and 80 mg/kg (1.83 ± 0.10 U/mg protein and 1.76 ± 0.17 U/mg protein, resp.). The results show that hippocampal iNOS levels of diabetic rats were correlated negatively with performance (Figure 8). These results suggest that the effect of saffron extract on the STZ-enhanced hippocampal iNOS levels might contribute to the amelioration of memory deficits in diabetic rats.

## 4. Discussion

There is clinical evidence that suggests that patients with diabetes have impaired cognitive functions but evidence from experimental studies is not clear. The potential mechanisms for this include direct effects of hypo- or hyperglycaemia and hypo- or hyperinsulinemia and also indirect effects via cerebrovascular alterations [20, 21]. This study analyzed the role of saffron on the biochemical and behavioral function of diabetic rats. The results of the present study indicate that intraperitoneal injection of saffron significantly ameliorated the adverse metabolic effects in rats treated with STZ. Experimental diabetes models can be induced by chemicals that selectively destroy the insulin-producing *β*-cells in the pancreas [22]. Streptozotocin-induced diabetes produced marked impairment in cognitive function which was coupled with marked increase in oxidative stress in the brain. Chronic treatment with saffron significantly ameliorated cognitive deficits. It has been shown that saffron can prevent the impairment of learning and memory to the hippocampus induced by chronic stress and also saffron and its active component (crocin) have the enhancing effects on memory and, therefore, demonstrate its implication in the mechanisms underlying recognition and spatial memory [23].

The direct glucose toxicity in the neurons is especially due to increased intracellular glucose oxidation [24], which leads to an increase in reactive species production [25]. Oxidative stress seems to play a central role in brain damage [26]. Recently, it has been reported that oxidative damage to rat synapses contributes to cognitive deficit [27]. Our results revealed that during experimental period, blood glucose level in untreated diabetic rats was significantly higher compared to the normal control rats. High blood glucose level causes deterioration of pancreatic *β* cells due to oxidative stress. The results showed that STZ induced diabetic rats had significantly higher serum levels of total lipids, TC, triglycerides, LDL as compared to the normal control group. Increased plasma lipid profile levels in diabetes may be related to the changes in lipid metabolism and structure. Recently, our research also demonstrated that plasma cholesterol and triglycerides levels were increased significantly in diabetic rats induced by STZ [22]. Previous study demonstrated that in diabetic rats the utilization of impaired carbohydrate leads to accelerate lipolysis, resulting in hyperlipidaemia and increased lipid peroxidation which is associated with hyperlipidaemia [28].

In the present study, diabetes caused significant increase in the level of blood glucose along with reduction in body weight. Saffron injection after STZ treatment resulted in lower serum glucose levels and improved lipid profile as well as body weight as compared with rats treated with STZ alone. Lozano et al. reported that saffron extract has various compounds like Krustyn, crocins including the crocin and tricrocin, pykrvkrvsyn, and safranal [29]. These active constituent have antioxidants properties which may be very important in mitigating impaired insulin secretion and action in insulin resistance and prevent diabetes complications [30]. The hypoglycemic effect of saffron extract seems to be exerted by mechanisms such as insulin resistance reducing [31], stimulating of glucose uptake by peripheral tissues [32], and inhibition of intestinal glucose absorption [33]. Regarding the hypolipidemic effects of saffron, Sheng et al., [34] indicated that crocin has lipid lowering properties and selectively inhibits the activity of pancreatic lipase as a competitive inhibitor. Moreover, He et al. [35] found that crocin has a potent hypotriglyceridemic and hypocholesterolemic activity in atherosclerotic quails, so that saffron is beneficial for curing of cardiovascular disorders [36].

The central nervous system (CNS) is sensitive to oxidative stress. Most of the reactive oxygen species- (ROS-) dependent central nervous disorders have been observed to be actually triggered by the presence of free radicals. Free radical generation during brief periods of cerebral ischaemia has been suggested to induce delayed neuronal death [37]. Antioxidant therapy has proved to be remarkably beneficial to combat ROS-induced injury in the CNS [38]. A number of investigations have been performed to indicate that brain loss is a consequence of both type I and type II diabetes [7, 39]. However, the exact mechanism(s) as to how diabetic conditions could affect brain activity remains to be fully characterized. Hyperglycemia increases the generation of free radicals by glucose autooxidation and the increment of free radicals may lead to brain cell damage. Therefore, diabetes-induced oxidative damage is responsible for the changes occurring in the activities of membrane-bound enzymes of significance leading to impaired neuronal activity. Besides the enhanced level of ROS, nitric oxide levels are also increased, and Mastrocola et al. [40] reported that the expression of mitochondrial nitric oxide synthase appears to be significantly increased in the brain mitochondria of diabetic rats with consequent nitric oxide hyperproduction: in those conditions, nitric oxide inhibits cytochrome c oxidase activity but might also act on other mitochondrial components, inhibiting other respiratory chain complexes by nitrosylation or oxidizing protein thiols [40, 41]. Beside its effects on the respiratory chain, nitric oxide may also contribute to cell damage in diabetes, modulating glucose entry into the cells. Indeed, nitric oxide has been shown to upregulate glucose transporters in neurons [42]. This activity has been regarded as protective in conditions (such as cerebral ischemia) in which the glucose supply to the brain is reduced [43]. Our results showed an increase in nitrite levels in hippocampus of diabetic rats and chronic treatment with saffron significantly decreased hippocampal nitrite levels because of its potential to inhibit expression of inducible nitric oxide synthase [44] and nitric oxide scavenging effects [45].

Increasing evidence indicates that factors such as oxidative and nitrosative stress, glutathione depletion, and impaired protein metabolism can interact in a vicious cycle which is central to pathogenesis of dementia. Our previous studies showed that diabetes induces oxidative stress [22]. In the present study, reduced glutathione, superoxide dismutase, and catalase activities were markedly reduced in the hippocampus of diabetic rats. Treatment with saffron returned the levels of reduced glutathione, superoxide dismutase, and catalase towards their control values. These protective effects of saffron against oxidative stress are in agreement with our previously published report [46]. Yoshino et al. [47] reported crocetin- (saffron's component) induced inhibition of cellular reactive oxygen species generation. In the present study saffron significantly ameliorated the cognitive impairment in diabetic rats. Thus, saffron may be preventing oxidative stress in hippocampal neurons and consequently may be improving synaptic plasticity. A transitory brain glutathione deficit results in selectively damaged in spatial memory [48]. Diabetes is chronic metabolic disorder conncted with reduction in glutathione levels which finally terminates to memory deficits in diabetic animals. Since, saffron could prevent intracellular reduced glutathione depletion may due to its antioxidant properties (our unpublished data), therefore, reversal of diabetic cognitive dysfunction could be observed. In the present study, significant decline in GSH level and antioxidant enzymes activity including SOD and CAT in the hippocampal tissue of rats reflects oxidative stress of hippocampus in experimental diabetes. These results are in line with the findings reported by Feillet-Coudray et al. [49], who observed that STZ-induced diabetes in rat accompanied with an increase in the susceptibility to lipid peroxidation. The data of our study also revealed that daily treatment of saffron extract markedly improves antioxidant status of the hippocampus tissue of rats with streptozotocin-induced diabetes as GSH level and antioxidant enzymes activities comprising SOD and CAT significantly increased. GSH (an important part of the nonenzymatic antioxidant system) is a major nonprotein thiol in living organisms, which plays a central role in coordinating the body's antioxidant defense processes. Perturbation of GSH status of a biological system can lead to serious consequences. Reduction in GSH stores of the hippocampus tissue of diabetic rats suggest that oxidative stress due to free-radical damage is one of the possible mechanisms in the pathophysiology of diabetic encephalopathy. The current investigation showed that the administration of the saffron extract caused the increased GSH levels. This indicates that in the presence of saffron extract there is an improvement in the oxidative stress. Increased oxidative stress in the hippocampus tissue of streptozotocin diabetic rats was similarly reported. This was said to be a contributory factor in the development of the complications of diabetes. SOD, CAT, and GSH constitute a mutually supportive team of defense against ROS. SOD is a metalloprotein and is the first enzyme involved in the antioxidant defense by lowering the steady-state level of O_2_^−^. In hyperglycaemia, glucose undergoes autooxidation and produces superoxide, and it produces free radicals that in turn lead to lipid peroxidation in lipoproteins. CAT is localized in the peroxisomes or the microperoxisomes, which catalyses the decomposition of H_2_O_2_ to water and oxygen and thus protects the cell from oxidative damage produced by H_2_O_2_. In our study, decline in the activities of these enzymes in the hippocampus tissue of streptozotocin-induced animals and attainment of near normalcy in saffron extract treated rats indicate that oxidative stress elicited in the hippocampus of diabetic rats had been nullified due to the effect of the extract. Thus, considering the pathophysiology of diabetes and the results presented in the present work, it is sensible to suggest that saffron ameliorates oxidative stress parameters in the hippocampus. In fact, ROS causes damage in the mitochondrial oxidative phosphorylation [50]. In addition, Bhattacharya et al. [51] reported decreased mitochondrial membrane potential, enhanced cytochrome c release, reciprocal regulation of the Bcl-2 family, and increases of caspases 3 and 9 in alloxan-induced diabetes. The authors also showed that treatment with D-saccharic acid 1,4-lactone, a derivative of D-glucaric acid which has antioxidant properties, counteracted these changes.

The accumulation of GSP levels (GSP) and AGEs, derived from the nonenzymatic glycation of reducing sugars and proteins, contributes to the development of diabetic encephalopathy [52]. The daily intake of 40 and 80 mg/kg saffron significantly increased body weight and decreased blood glucose, GSP levels, and serum AGEs levels in STZ-induced diabetic encephalopathy rats. The findings indicate that saffron has a beneficial effect on diabetic encephalopathy. AGEs are the final products of nonenzymatic glycation and oxidation of proteins and lipids, which have been found to deposit in human tissues with aging and brains of diabetic patients [53]. AGEs are believed to play a crucial role in the development and the progression of diabetic encephalopathy by the damaging the structure and functional properties of the hippocampus and cerebral cortical neurons via binding to cell surface receptors for AGEs (RAGE) [15]. The receptor for AGEs (RAGE) is a multiligand receptor protein thought to play an important role in neuronal damage [54]. The interaction between AGEs and RAGE activates a variety of signaling pathways that lead to the pathogenesis of diabetic encephalopathy, such as neurodegeneration [55]. Our study also provided further confirmation that the accumulation of AGEs activated RAGE in the hippocampus neurons. However, saffron extract treatment ameliorated the elevated AGEs associated with diabetic encephalopathy. These results suggest that the decreased hyperglycemia with saffron administration might contribute to the amelioration of hyperglycemia-induced encephalopathy damage.

Proinflammatory cytokines (such as TNF-*α*) are known to be increased in several neuropathological states that are associated with learning and memory. In addition to oxidative and nitrosative stress, hyperglycemia is also associated with enhanced inflammatory response [56, 57]. Under chronic hyperglycemia, endogenous TNF-*α* production is in increased microvascular and neural tissues, which may cause accelerated microvascular permeability, hypercoagulability, and nerve damage, thus initiating and promoting the development of characteristic lesions of diabetic microangiopathy, polyneuropathy, and encephalopathy [58, 59]. It has been recently reported that uncontrolled diabetes significantly enhanced TNF-*α* level [60]. A significant inhibition of TNF-*α* levels by saffron observed in our study is indicative of the fact that saffron contributes to beneficial effects seen in diabetic encephalopathy. With respect to inflammation, saffron reduces the production of proinflammatory cytokines such as TNF-*α*.

In our knowledge for the first time, we showed the effects of the animal model of diabetes induced by STZ on oxidative stress parameters in the hippocampus of rats and also indicated for the first time that the treatment with saffron ameliorated them. The present study showed pharmacologic effect of saffron (*C. sativus* Linn.) extract in hippocampus complications of diabetes. Thus, saffron treatment ameliorated blood glucose, cognitive deficit, lipid profile, reduced oxidative stress, nitric oxide, and TNF-*α* in the diabetic rats. Moreover, the findings of the study also suggested that the principle mechanisms involved in the antidiabetic and neuroprotective effect of saffron are its strong antioxidant and anti-inflammatory potential. Although, its low side-effect profile and long history of safe use, many questions related to antihyperglycemic and antioxidant effect of* C. sativus* extract remain unanswered. Much more work is clearly needed before phytotherapy for diabetic encephalopathy can be advanced to clinic.

Therefore, it seems that saffron extract has positive effects on the prevention of early hippocampal injury in diabetes mellitus due to oxidative stress and can be recommended in diabetic humans as herbal drug after randomized clinical trials.

## Figures and Tables

**Table 1:** Effect of saffron extract on body weight in STZ-treated diabetic rats. Control (C), untreated diabetic rats (D), saffron extract (20 mg/kg/day)—treated diabetic rats (L-saf), saffron extract (40 mg/kg/day)—treated diabetic rats (M-saf), and saffron extract (80 mg/kg/day)—treated diabetic rats (H-saf) during 4 weeks of study.

Days	0	7	14	21	28
C					
Body weight (g)	199 ± 10	240 ± 12	257 ± 14	276 ± 16	301 ± 18
D					
Body weight (g)	215 ± 13	210 ± 11∗∗∗	201 ± 12∗∗∗	192 ± 10∗∗∗	183 ± 11∗∗∗
L-saf					
Body weight (g)	188 ± 12	184 ± 7∗∗	180 ± 9∗∗	187 ± 13∗∗	194 ± 15∗∗
M-saf					
Body weight (g)	208 ± 11	211 ± 12∗	220 ± 14∗	226 ± 13^∗∗,+^	231 ± 12^∗∗,+^
H-saf					
Body weight (g)	199 ± 9	203 ± 10	216 ± 12	228 ± 18^+^	239 ± 14^++^

Each measurement was done at least in triplicate and the values are the means 00B1 SEM for eight rats in each group.

Significantly different from normal control (Group C) rats (*P < 0.05, **P < 0.01, ***P < 0.001).

Significantly different from STZ-treated (Group D) rats (^+^ *P* < 0.05, ^++^ *P* < 0.01).
