# Corrigendum to “Ameliorative Effect of Saffron Aqueous Extract on Hyperglycemia, Hyperlipidemia, and Oxidative Stress on Diabetic Encephalopathy in Streptozotocin Induced Experimental Diabetes Mellitus”

**DOI:** 10.1155/2020/7949263

**Published:** 2020-11-22

**Authors:** BioMed Research International

The article titled “Ameliorative Effect of Saffron Aqueous Extract on Hyperglycemia, Hyperlipidemia, and Oxidative Stress on Diabetic Encephalopathy in Streptozotocin Induced Experimental Diabetes Mellitus” [1] was found to contain material from published work by other authors that was not cited [2]. The copied article is as follows:

Anurag Kuhad and Kanwaljit Chopra, “Curcumin attenuates diabetic encephalopathy in rats: behavioral and biochemical evidences,” European Journal of Pharmacology, Volume 576, Issues 1–3, 2007, Pages 34-42, https://www.sciencedirect.com/science/article/pii/S0014299907008916

Some of the text in the “Materials and Methods” were reused, specifically about the “Morris Water Maze Test” and “Estimation of Tumor Necrosis Factor-Alpha (TNF-*α*),” and there was also reuse of text in the “Discussion”: many sentences in the first paragraph, the first two sentences in the second paragraph, the second half of the fourth paragraph, many sentences at the beginning of the fifth paragraph, and most of the seventh paragraph.

The authors said a translation company to which they sent the article may have used wording from other related sources and do not agree with the publication of this corrigendum.
